# Exogenous IL‐6 induces mRNA splice variant MBD2_v2 to promote stemness in TP53 wild‐type, African American PCa cells

**DOI:** 10.1002/1878-0261.12316

**Published:** 2018-05-24

**Authors:** Emily A. Teslow, Bin Bao, Greg Dyson, Christophe Legendre, Cristina Mitrea, Wael Sakr, John D. Carpten, Isaac Powell, Aliccia Bollig‐Fischer

**Affiliations:** ^1^ Barbara Ann Karmanos Cancer Institute Detroit MI USA; ^2^ Department of Oncology Wayne State University School of Medicine Detroit MI USA; ^3^ Integrated Cancer Genomics Division Translational Genomics Research Institute Phoenix AZ USA; ^4^ Department of Computer Science Wayne State University Detroit MI USA; ^5^ Department of Pathology Wayne State University School of Medicine Detroit MI USA; ^6^ Department of Translational Genomics Keck School of Medicine University of Southern California Los Angeles CA USA; ^7^ Department of Urology Wayne State University School of Medicine Detroit MI USA

**Keywords:** African American, cancer stem‐like cells, IL‐6, MBD2, mRNA splice variant, prostate cancer

## Abstract

African American men (AAM) are at higher risk of being diagnosed with prostate cancer (PCa) and are at higher risk of dying from the disease compared to European American men (EAM). We sought to better understand PCa molecular diversity that may be underlying these disparities. We performed RNA‐sequencing analysis on high‐grade PCa to identify genes showing differential tumor versus noncancer adjacent tissue expression patterns unique to AAM or EAM. We observed that interleukin‐6 (IL‐6) was upregulated in the nonmalignant adjacent tissue in AAM, but in EAM IL‐6 expression was higher in PCa tissue. Enrichment analysis identified that genes linked to the function of TP53 were overrepresented and downregulated in PCa tissue from AAM. These RNA‐sequencing results informed our subsequent investigation of a diverse PCa cell line panel. We observed that PCa cell lines that are TP53 wild‐type, which includes cell lines derived from AAM (MDA‐PCa‐2b and RC77T), did not express detectable IL‐6 mRNA. IL‐6 treatment of these cells downregulated wild‐type TP53 protein and induced mRNA and protein expression of the epigenetic reader methyl CpG binding domain protein 2 (MBD2), specifically the alternative mRNA splicing variant MBD2_v2. Further investigation validated that upregulation of this short isoform promotes self‐renewal and expansion of PCa cancer stem‐like cells (CSCs). In conclusion, this report contributes to characterizing gene expression patterns in high‐grade PCa and adjacent noncancer tissues from EAM and AAM. The results we describe here advance what is known about the biology associated with PCa race disparities and the molecular signaling of CSCs.

**Abbreviations**
AAM: African American men
CSC: cancer stem‐like cell
EAM: European American men
FFPE: formalin‐fixed paraffin‐embedded
FPKM: fragments per kilobase of exon per million reads
IL‐6: interleukin‐6
MBD2: methyl CpG binding domain protein 2
NuRD: nucleosome remodeling and deacetylase
PCa: prostate cancer
ROS: reactive oxygen species

## 1 Introduction

There are approximately 160 000 new cases of prostate cancer (PCa) and 26 730 PCa‐related deaths annually in the United States (American Cancer Society, 2017), making PCa the second leading cause of cancer‐related deaths for American men. Recent statistics also reveal that race disparities persist despite improvements in PCa detection, access to care, and survival across all demographics (American Cancer Society, 2016, 2017; Powell *et al*., 2010). African American men (AAM) have a 70% higher incidence rate and a twofold to fivefold greater risk of dying from the disease compared to European American men (EAM) (American Cancer Society, 2016). Moreover, AAM diagnosed with low‐risk prostate cancer are more likely to harbor higher risk disease (Maurice *et al*., 2017). The cause of these disparities is likely multifaceted, including undetermined contributions from ancestry genetics and lifestyle risk factors (Barrington *et al*., 2015; Cooperberg, 2013; Hsing *et al*., 2007; Major *et al*., 2012; Parker *et al*., 2011), and reviewed in Powell and Bollig‐Fischer (2013). This raises the fundamental motivation for our work: that the molecular underpinnings for race disparities in PCa, which remain to be understood, may one day be exploited to advance clinical decision‐making and improve outcomes for all patients.

Traditionally, AAM have been poorly represented in reports of molecular genomic aberrations in PCa. Recent research has begun to address this shortcoming and to highlight the greater molecular complexity of the disease. Most notably, it is validated that TP53 somatic aberrations and TMPRSS2‐ERG fusions occur significantly less often in tumors from AAM relative to EAM (Huang *et al*., 2017; Khani *et al*., 2014; Lindquist *et al*., 2016; Tomlins *et al*., 2015; Yamoah *et al*., 2015). Amplification of the fatty acid synthase (FASN) gene, however, was found to be more frequent in PCa samples from AAM (Huang *et al*., 2017), and this is consistent with our finding that FASN mRNA expression is increased in PCa from AAM relative to EAM (Powell *et al*., 2013).

In the current study, we performed RNA‐sequencing analysis to further understand the molecular diversity of PCa by specifically investigating high‐grade PCa [Gleason Score (GS) ≥ 7(4 + 3)] in relation to matched noncancer adjacent tissue across AAM and EAM. Here, our RNA‐sequencing data analysis identified cytokine signaling factors including interleukin‐6 (IL‐6) as showing race‐specific differential expression. For AAM, IL‐6 was upregulated in the nonmalignant adjacent tissue, but for EAM, IL‐6 expression was higher in PCa tissue. Much effort has been put forth to study the mechanistic role of IL‐6 in PCa, supporting that IL‐6 is a key cancer‐promoting factor and rational therapeutic target (Lee *et al*., 2003; Qu *et al*., 2013; Zhong *et al*., 2016). However, this narrative is challenged by reports such as one from Pencik *et al*. (2015) showing that STAT3 activation, the downstream effector of IL‐6 signaling, suppresses PCa progression (Pencik *et al*., 2015). Moreover, clinical trials using antibodies to target IL‐6 failed to provide benefit to PCa patients (Dorff *et al*., 2010; Fizazi *et al*., 2012). Yet, increased levels of IL‐6 in patient serum associated with poor outcomes (Nakashima *et al*., 2000), and serum IL‐6 levels are known to be higher in AAM than in EAM (Paalani *et al*., 2011). The importance of IL‐6 in PCa race disparities remains unresolved.

The RNA‐sequencing results that we report herein associated with AAM led us to recognize the potential for microenvironment‐derived (exogenous) IL‐6 to inactivate tumor suppressor TP53 in PCa cells that do not express IL‐6. Using a panel of PCa cells, including cell lines from AAM, we also elucidated that exogenous IL‐6 upregulated expression of the epigenetic reader methyl CpG binding domain protein 2 (MBD2), specifically the alternative mRNA splicing variant MBD2_v2, to promote cancer stem‐like cells (CSCs). The work we describe advances what is known about the biology associated with PCa race disparities and molecular signaling promoting CSCs.

## 2 Materials and methods

### 2.1 RNA sequencing of patient samples

Specimen collection and analysis were carried out with the understanding and written consent of each subject. The study methodologies conformed to the standards set by the Declaration of Helsinki and were approved by the Wayne State University Institutional Review Board. RNA sequencing was applied to matched PCa and adjacent noncancer prostate tissue specimens from 16 patients, eight AAM and eight EAM, for a total of 32 samples. All PCa specimens represented an aggressive phenotype, with GS ≥ 7(4 + 3) (Stark *et al*., 2009). De‐identified, formalin‐fixed paraffin‐embedded (FFPE) high‐grade PCa (greater than 70% cancer cell content) and matched adjacent nonmalignant tissue samples were identified and reviewed at the Biorepository in the Department of Pathology at Wayne State University, Detroit, MI. Total RNA was isolated from the FFPE specimens (eight sections, 10 μm each per block; discarding surface section) using the Recover All kit for FFPE, with extended proteinase K and DNAse treatment (Life Technologies Inc., Carlsbad, CA, USA). RNA quantity and quality were estimated by spectrophotometry. Double‐stranded cDNA preparation and library construction were done with the Ovation Human FFPE RNA‐Sequencing Multiplex System (NuGEN, San Carlos, CA, USA) using 200 ng total RNA. Key features are as follows: it is strand‐specific; no poly‐A selection step (or other selection step that could introduce bias or be problematic for degraded RNA); and the approach integrates an insert dependent adapter cleavage step that specifically targets ribosomal RNA for degradation (Adiconis *et al*., 2013). Quality of library preparations was assessed using the Tapestation (Agilent, Santa Clara, CA, USA) (Fig. S1). Cluster generation was performed using the Illumina cBot and HiSeq Paired End Cluster Generation Kit (Illumina, San Diego, CA, USA). Flow cells were paired end sequenced (100 cycles) on an Illumina HiSeq 2500 (high‐output mode). Sample libraries were indexed and multiplexed in randomized fashion: four per lane of an 8‐lane flow cell. FastQC analysis (http://www.bioinformatics.babraham.ac.uk/) was done to know that more than 85% of reads, for all samples, passed QC30. Transcript and gene‐level expression abundances were calculated using the cufflinks2 module from the Cufflinks2 Suite (Trapnell *et al*., 2009, 2012). The abundance results were reported in plain text files showing *P*‐values (adjusted for multiple testing) and normalized abundance data in terms of FPKM (fragments per kilobase of transcript per million mapped reads).

In an additional quality control step, we ran a test of the nonparametric Spearman correlation between identical samples sequenced twice, in different batches, which demonstrated high reproducibility (98%, data not shown). We also compared our RNA‐sequencing data with expression data from our published study that employed microarray‐based analysis (Powell *et al*., 2013). Applying nonparametric Spearman correlation analysis to measurements from the two technologies yielded a high correlation (0.805 AAM and 0.811 EAM, Fig. S2), signifying that the results of high‐throughput sequencing compared to gene expression measured by validated microarray analysis across the bulk of genes analyzed by both methods, even though the PCa samples studied were different.

### 2.2 Statistical analysis of RNA‐sequencing data

Matched high‐grade [GS ≥ 7(4 + 3)] prostate tumor and adjacent normal specimens from 16 patients (eight AAM and eight EAM, Table S1) were subjected to two replicate runs of RNA‐sequencing analyses. The standard fragments per kilobase of exon per million reads (FPKM) per transcript were normalized by adding 1 and applying a log‐transformation. A mixed model analysis was used to model normalized read count as a function of race, tissue type (tumor or normal), and their interaction for each transcript, accounting for the correlation between replicates and different variance in the two batches. The outcomes identified transcripts with a significant (*P* ≤ 0.05) interaction effect between race and tissue type. FASTQ and processed data are available at Gene Expression Omnibus GSE104131. The Enrichr tool (Kuleshov *et al*., 2016) was applied to the resulting significant gene list to identify significantly overrepresented KEGG pathways (*P* ≤ 0.05). The Upstream Regulator analysis tool (Krämer *et al*., 2014), included in the Ingenuity Systems (Qiagen, Redwood City, CA, USA) software suite, was used to identify significant overenrichment (*P* ≤ 0.05) for subsets of genes associated with activation or inactivation of upstream regulators.

### 2.3 Cell lines, viability assays, real‐time RT–PCR, and immunoblot analysis

Details on cell line authentication, cell culture media conditions, methods for viability, RT–PCR, and immunoblot analyses are presented in Data S1.

### 2.4 Prostasphere formation assay

The presence of and self‐renewal capacity of CSCs was examined in PCa cell lines by this sphere‐propagating assay as described previously (Bao *et al*., 2017; Rybak *et al*., 2011). Briefly, 1000 single cells were seeded in 1.5 mL of the FBS‐free sphere formation media (1 : 1 DMEM:F‐12 media plus with B‐27 and N‐2 supplements, Gibco Brand, ThermoFisher, Waltham, MA, USA) in six‐well Ultra Low Attachment plates (Corning Inc., Corning, NY, USA). Treatments were added and media replenished every 3 days. After 7 days of incubation, the prostaspheres (at a size equal or greater than 50 μm diameter) were counted and reported as a fraction of the total number of cells seeded. Images were taken using a Nikon Eclipse TE2000‐U microscope at 40× magnification (Tokyo, Japan).

### 2.5 Fluorescence‐activated cell sorting (FACS) analysis

CSCs and total PCa cells were counted by fluorescence‐activated cell sorting (FACS) analysis, using the BD LSR II (BD Biosciences, San Jose, CA, USA), at the Karmanos Cancer Institute Microscopy, Imaging and Cytometry Resources Core. CSCs were sorted based on triple‐marker (CD44+/CD133+/EpCAM+)‐positive status. Fluorochrome‐labeled monoclonal antibodies against human CD44, CD133, and EpCAM proteins were obtained from EBiosciences (San Diego, CA, USA; catalogue number 25‐0441‐82), Miltenyil Biotec (Cologne, Germany; catalogue number 130‐090‐854), and BD Biosciences (Franklin Lakes, NJ, USA; catalogue number 347198), respectively.

### 2.6 Stable overexpression of MBD2_v2 in prostate cancer cell lines

Packaged lentiviral particles to overexpress GFP or mCherry control genes, or MBD2_v2 were purchased from Cyagen Biosciences (Santa Clara, CA, USA). The custom‐synthesized human MBD2_v2 (NM015832.4) gene, mCherry, or GFP sequence was subcloned into a lentiviral expression vector downstream of the CMV promoter. The construct was sequenced to ensure that the MBD2_v2 sequence and orientation were correct. The expression vector also expressed a puromycin resistance gene. Cells were transduced and selected with puromycin. GFP expression was visible by fluorescence microscopy. Overexpression of MBD2_v2 was validated by immunoblot analysis and semiquantitative RT–PCR using TaqMan probes.

### 2.7 Meta‐analysis of MBD2_v2 expression using the Oncomine database

Microarray data from the Oncomine database was accessed on May 22, 2017 (Rhodes *et al*., 2004). All PCa datasets utilizing the splice variant‐specific Affymetrix probe for MBD2_v2 (214396_s_at) or MBD2_v1 (202484_s_at), were queried to obtain log_2_ median‐centered intensities, based on GS for clinical specimens only. Patient specimens (*n *=* *244) from a total of five studies (Best *et al*., 2005; Glinsky *et al*., 2004; Liu *et al*., 2006; Vanaja *et al*., 2003; Wallace *et al*., 2008), were partitioned into two groups representing low‐grade and high‐grade PCa (GS 4‐7 and GS 8‐9). A two‐sided unpaired *t*‐test was performed on log_2_ median‐centered intensities to compare the two groups.

### 2.8 Statistical analysis of data from PCa cell line experiments

Statistical analysis of data resulting from experiments using PCa cell lines was performed using graphpad prism (GraphPad Software Inc., La Jolla, CA, USA). Semiquantitative RT–PCR data are presented as the mean and standard deviation of a representative experiment. Mann–Whitney *U* test or unpaired two‐sided *t*‐test (Welch's *t*‐test) was performed to test the significance of difference between two groups, a *P*‐value ≤ 0.05 is considered to be statistically significant.

## 3 Results

### 3.1 RNA‐sequencing analysis of PCa and noncancer prostate tissue from AAM and EAM

**Figure 1:**
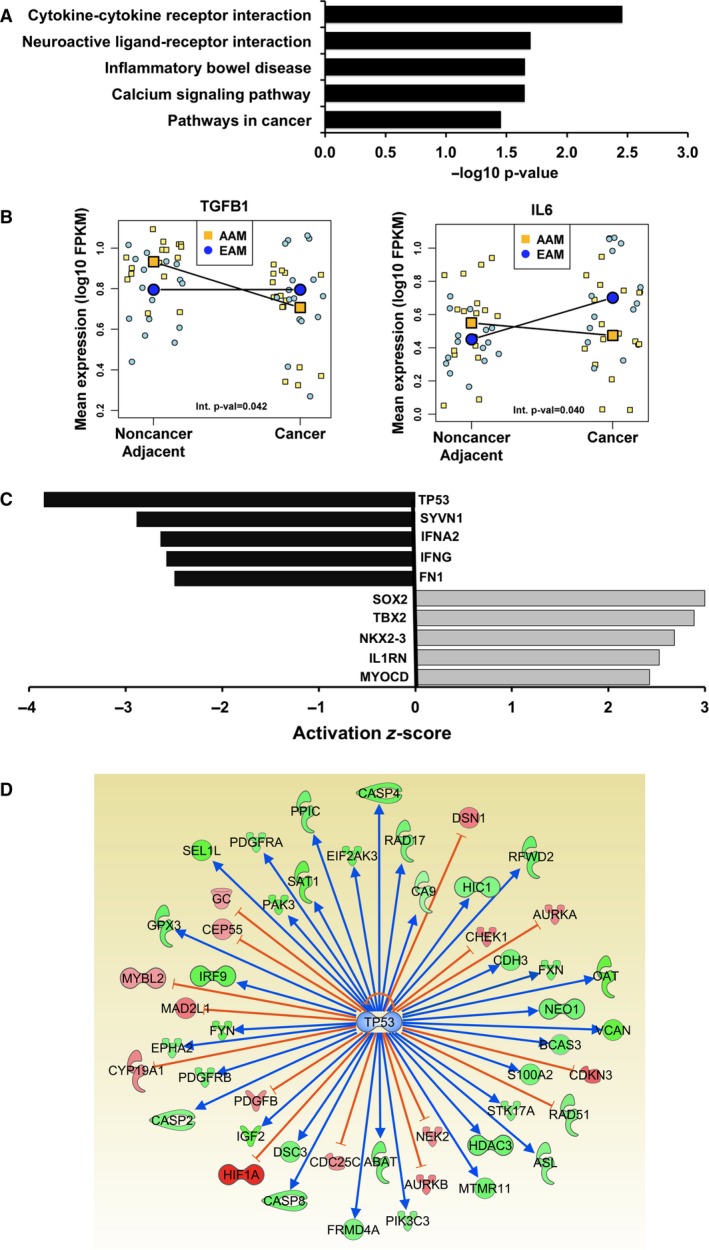
Analysis of RNA‐sequencing data from PCa and matched noncancer adjacent tissue identified race‐specific differential gene expression. RNA sequencing and interaction effect analysis were run on PCa and matched noncancer adjacent tissues from eight AAM and eight EAM (32 samples total, repeated). (A) The Enrichr tool was applied to the resulting significant gene set to identify KEGG signaling pathways overrepresented in the data. (B) Race‐specific, differential gene expression patterns are shown for TGFB1 and IL‐6, which were among the genes contributing to significant overrepresentation of the cytokine–cytokine receptor interaction pathway in (A). The interaction effect analysis *P*‐value is provided. (C) Upstream Regulator analysis compared our input list of differentially expressed genes to a catalogue of perturbed datasets to consider the significance of gene overlap and direction of expression differences to predict the activity of upstream regulators, for example, transcription factors. The algorithm accounts for the direction of differential expression of genes downstream of an upstream regulator to calculate a negative activation *z*‐score (predictive of inactivation) or a positive activation *z*‐score (predictive of activation). (D) Enriched network of genes associated with TP53 function identified by Upstream Regulator Analysis. The patterns of expression displayed here represent PCa relative to noncancer adjacent tissues specific to AAM. Green nodes showed significant (*P* ≤ 0.05) decreased expression, and red nodes were significantly increased. The edges connecting TP53 to other genes represent published regulatory relationships: blue activating expression and orange inhibitory. The result indicates that although TP53 mRNA levels were not different for either EAM or AAM, TP53 function was being inactivated in PCa from AAM.

We analyzed RNA‐sequencing normalized read count differences between tumor and adjacent nonmalignant tissue samples as a function of race. Plots for the nine most significant differentially expressed genes among the resulting 1206 significant coding genes identified are provided in Fig. S3. We then applied the Enrichr tool (Kuleshov *et al*., 2016) to the significant gene set to identify significant signaling pathways overrepresented in the data. Cytokine–cytokine receptor interaction was the most significant pathway (Fig. 1A). The genes associated with this pathway in our dataset are provided in Table S2. Among them, IL‐6 and TGFB1 were upregulated in the noncancer, tumor‐adjacent tissue of AAM, but for EAM, IL‐6 expression was increased in PCa tissue and TGFB1 was not differentially expressed (Fig. 1B). We further examined our significant gene set using the Upstream Regulator tool (Krämer *et al*., 2014). Upstream Regulator analysis compared our input list of differentially expressed genes to a catalogue of perturbed datasets to consider the significance of gene overlap and direction of expression differences to predict the activity of upstream regulators. This revealed a significant overrepresentation and coordinated change in mRNA expression in AAM tumor data for genes that are known to be regulated by tumor suppressor protein TP53 (Fig. 1C). Specifically, the direction of differential expression of genes downstream of TP53 suggested that TP53 inactivation was occurring in PCa from AAM (Fig. 1D).

### 3.2 IL‐6 treatment promotes CSC growth in IL‐6 nonexpressing PCa cell cultures

Our RNA‐sequencing analysis of high‐grade PCa and noncancer adjacent tissues revealed differential IL‐6 expression specific to race (Fig. 1). The data from AAM suggest a paracrine role for IL‐6, but IL‐6 expression was enriched in PCa specimens from EAM, indicating that for some high‐grade tumors, PCa cells may express autocrine‐acting IL‐6. We set out to further distinguish the role of IL‐6 using a diverse panel of PCa cells, including cell lines from AAM. We began by characterizing IL‐6 expression levels. Based on results of real‐time RT–PCR analysis using TaqMan probes, IL‐6 mRNA was not detected in MDA‐PCa‐2b, RC77T or LNCaP cells, but it was highly expressed in PC3 and DU145 cells. The results in Table 1 are annotated with the information that MDA‐PCa‐2b and RC77T were derived from PCa from AAM. Also, the cell lines expressing IL‐6 are TP53 mutant. IL‐6 mRNA was not detected in TP53 wild‐type cell lines (Table 1).

**Table 1:** PCa cell lines differ in endogenous IL‐6 expression. IL‐6 levels were measured in PCa cell lines by real‐time RT–PCR analysis using TaqMan probes. Relative levels for PC3 and DU‐145 cells, which expressed IL‐6, were calculated by the ΔΔ*C*_t_ method using βactin expression as the normalizer. Cell lines were authenticated, and the TP53 mutation status according to the COSMIC database is listed

PCa cell line	Relative IL‐6 expression level	TP53 mutations
PC3	6.18	p.138fs
DU‐145	1.00	p.V274F
LNCaP	Not detected	Wild‐type
MDA‐PCa‐2b^a^	Not detected	Wild‐type
RC77T^a^	Not detected	Wild‐type

Samples were determined Not Detected by the One Step Plus Systems (Applied Biosystems, Foster City, CA, USA).

a Derived from tumors from AAM.

**Figure 2:**
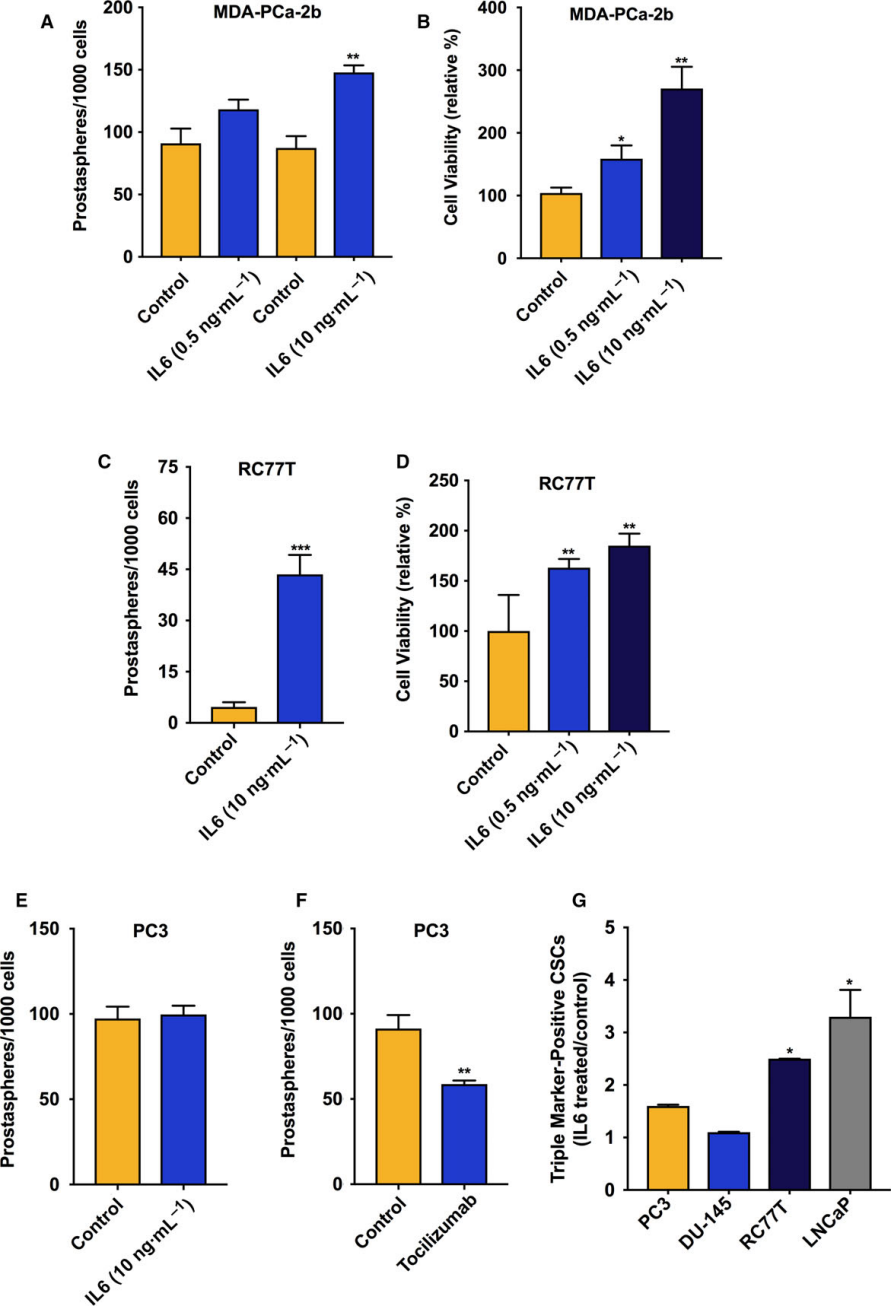
IL‐6 treatment induced prostasphere formation in IL‐6 nonexpressing PCa cell line cultures. (A) Effect of IL‐6 treatment relative to vehicle control on the numbers of prostaspheres in 7‐day cultures of MDA‐PCa‐2b cells. (B) Effect of IL‐6 treatment (72‐h) on viability of MDA‐PCa‐2b cells, run in triplicate and repeated twice. (C) Effect of IL‐6 treatment relative to vehicle control on the numbers of prostaspheres in 7‐day cultures of RC77T cells. (D) Effect of IL‐6 on viability of RC77T cells, 7‐day treatment run in triplicate and repeated twice. (E) Effect of 7‐day IL‐6 treatment on the numbers of PC3 prostaspheres. (F) Effect of IL‐6 receptor inhibitor tocilizumab (10 μm, 7 days) on prostaspheres in PC3 cultures. (G) Impact of IL‐6 treatment on the percentage of CSCs in other cell lines in our panel measured by FACS analysis. Cells were treated with IL‐6 at 10 ng·mL^−1^ for 7 or 14 days for RC77T. The fraction of CSCs relative to total cell count was measured based on CSC triple‐marker‐positive status (CD44+/CD133+/EpCAM+). The results are presented as fold‐change, IL‐6 treated vs. control. Prostasphere assay and FACS data are representative of repeated experiments and are the average of three independent biological replicates. *P ≤ 0.05, **P ≤ 0.01.

It was previously reported that IL‐6 signaling in PCa sustains and promotes the generation of CSCs (Kroon *et al*., 2013; Qu *et al*., 2013). We proceeded to measure the impact that IL‐6 had on promoting CSCs across our PCa cell line panel. Using a prostasphere formation assay, we tested whether IL‐6 treatment influenced the formation of prostaspheres, demonstrating the presence of CSCs (Rybak *et al*., 2011). In the AA‐derived MDA‐PCa‐2b cells, which do not express IL‐6, we observed an increase in the number of prostaspheres after 7 days of low‐dose IL‐6 treatment, and a higher IL‐6 concentration elicited a more significant increase in the number of prostaspheres (Fig. 2A). We further observed that within 48 h MDA‐PCa‐2b cell viability also increased in a dose‐dependent manner (Fig. 2B). We then tested IL‐6 treatment on cultures of RC77T cells, which are also of AA origin and TP53 wild‐type, and do not express IL‐6. Similar to MDA‐PCa‐2b cells, IL‐6 treatment induced greater numbers of prostaspheres (Fig. 2C) and increased cell viability similar to MDA‐PCa‐2b cells (Fig. 2D). For PC3 cells, which are TP53 mutant and express high levels of IL‐6 endogenously, IL‐6 treatment had no effect on prostasphere growth (Fig. 2E). However, treatment of PC3 cells with the IL‐6 receptor inhibitor tocilizumab reduced prostasphere formation (Fig. 2F).

The impact of IL‐6 on CSCs on other prostate cancer cell lines in our panel was measured by FACS, where the fraction of CSCs was measured based on triple‐marker‐positive status (CD44+/CD133+/EpCAM+). This assay distinguishes CD133‐positive CSCs relative to non‐CSCs, also referred to as bulk cancer cells, that do not express CD133. CD133 is a specific PCa CSC surface marker (Richardson *et al*., 2004). For IL‐6 expressing DU145 cell line cultures, IL‐6 treatment for 7 days had no effect on the fraction of triple‐marker‐positive cell numbers. However, for IL‐6 nonexpressing LNCaP cells, a similar 7‐day IL‐6 treatment regimen induced a threefold increase in the percentage of triple‐marker‐positive CSCs (Fig. 2G, Table S3). Results using PC3 again showed that IL‐6 treatment had no effect on prostaspheres (Fig. 2G). For IL‐6 nonexpressing RC77T cells, an increase in the percentage of triple‐marker‐positive CSCs was significant at 14 days of treatment (Fig. 2G, Table S3).

### 3.3 IL‐6 treatment induced expression of alternative mRNA splicing variant MBD2_v2, which promotes CSCs

**Figure 3:**
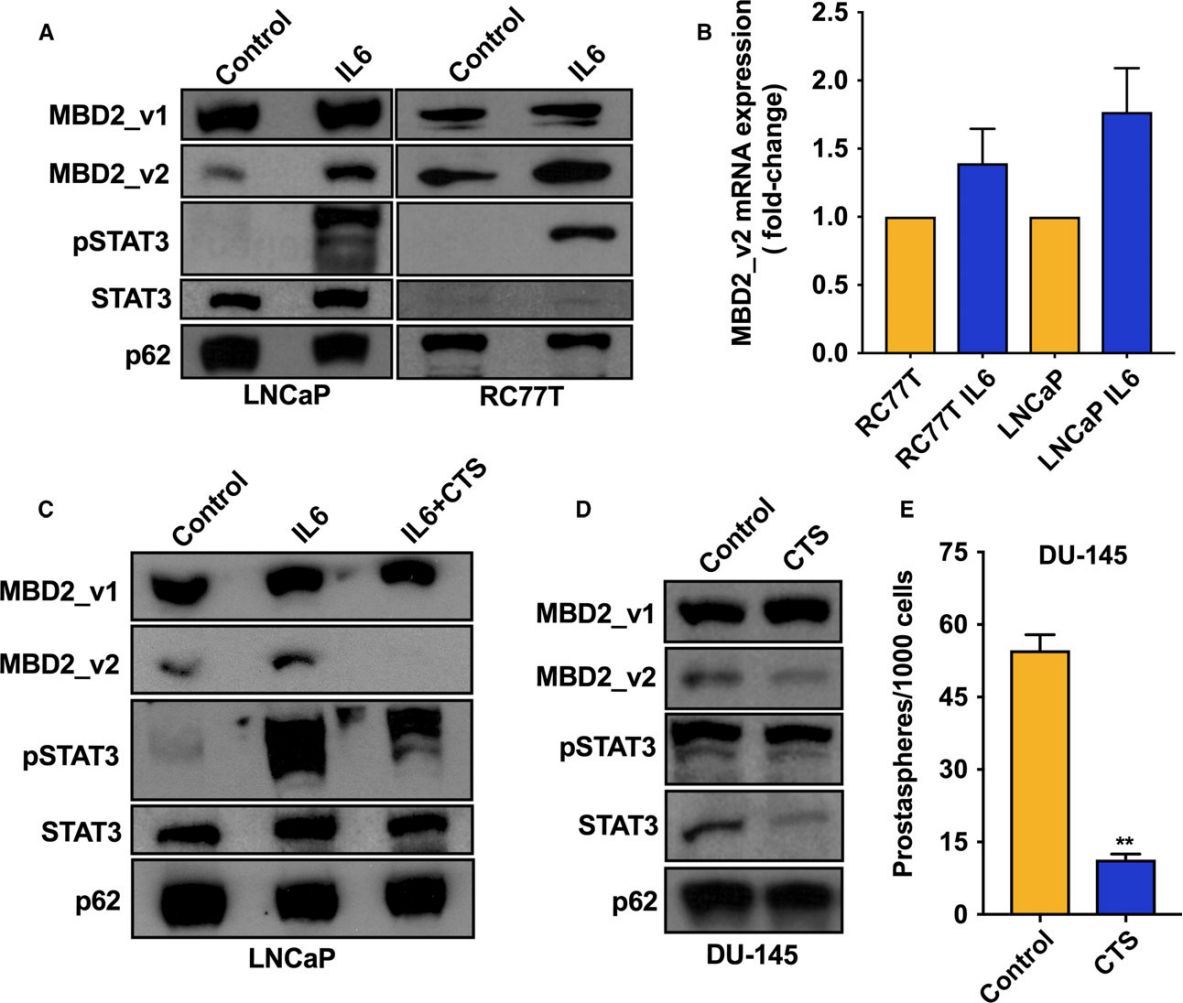
Activation of IL‐6 signaling upregulated expression of the MBD2 short isoform MBD2_v2 in PCa cell lines. (A) Immmunoblot analysis of MBD2 isoforms, phosphorylated STAT3 (pSTAT3), and total STAT3 protein levels in IL‐6 nonexpressing cell lines LNCaP and RC77T treated with IL‐6 (10 ng·mL^−1^, 14 days) or diluent control. (B) MBD2_v2 mRNA levels in LnCaP and RC77T cell lines measured by real‐time RT–PCR using TaqMan probes. Results are presented as fold‐change, IL‐6‐treated relative to vehicle‐treated conditions. (C) Immunoblot analysis of MBD2 isoforms, pSTAT3, and total STAT3 protein in LNCaP cells treated with IL‐6 in combination with the STAT3 inhibitor drug cryptotanshinone (CTS, 500 nm) or vehicle control for 14 days. (D) Immunoblot analysis of MBD2 isoforms, pSTAT3, and total STAT3 protein in IL‐6‐expressing cell line DU‐145, treated with CTS (500 nm) or vehicle control for 48 h. Cell culture treatment, protein harvest, and immunoblot analysis were carried out three times. (E) Effect of CTS treatment relative to vehicle control on the numbers of prostaspheres in 7‐day cultures of DU145 cells. **P ≤ 0.01.

We recently identified in triple negative breast cancer, an aggressive breast cancer subtype that disproportionately affects African American women (Brewster *et al*., 2014), that expression of epigenetic reader methyl binding domain protein 2 (MBD2), specifically the alternative mRNA splicing variant MBD2_v2, is dependent on reactive oxygen species (ROS) and necessary to maintain the cancer stem cell phenotype (Bao *et al*., 2017). In generating PCa CSCs, IL‐6 activity is coupled with the production of ROS, which function as second messenger signaling factors (Qu *et al*., 2013). Therefore, we hypothesized that IL‐6 treatment of PCa cells upregulates expression of MBD2_v2 and that increased MBD2_v2 expression promotes PCa CSCs. We tested this using IL‐6 nonexpressing RC77T and LNCaP cells. As can be seen from immunoblot analysis, IL‐6 treatment induced increased protein and mRNA expression of the MBD2_v2 isoform in both cells lines (Fig. 3A,B). Levels of the long isoform, mRNA variant MBD2_v1, were not affected by IL‐6 treatment (Fig 3A). The addition of a pharmacological STAT3 inhibitor blocked IL‐6 induction of MBD2_v2 (Fig. 3C), corroborating the role of exogenous IL‐6 signaling via STAT3. Treatment with a STAT3 inhibitor alone downregulated MBD2_v2 (Fig. 3D), and prostaspheres (Fig. 3E) in IL‐6 expressing DU145 cells, indicating that MBD2_v2 levels and prostaspheres were sustained by the endogenous IL‐6 signaling in this cell line. Regarding STAT3 immunoblotting, each of the panels (Fig. 3A,C,D) demonstrate that STAT3 phospho‐protein levels (pSTAT3) were induced by IL‐6 treatment, while total protein levels were unaffected, which is consistent with canonical IL‐6 signaling.

**Figure 4:**
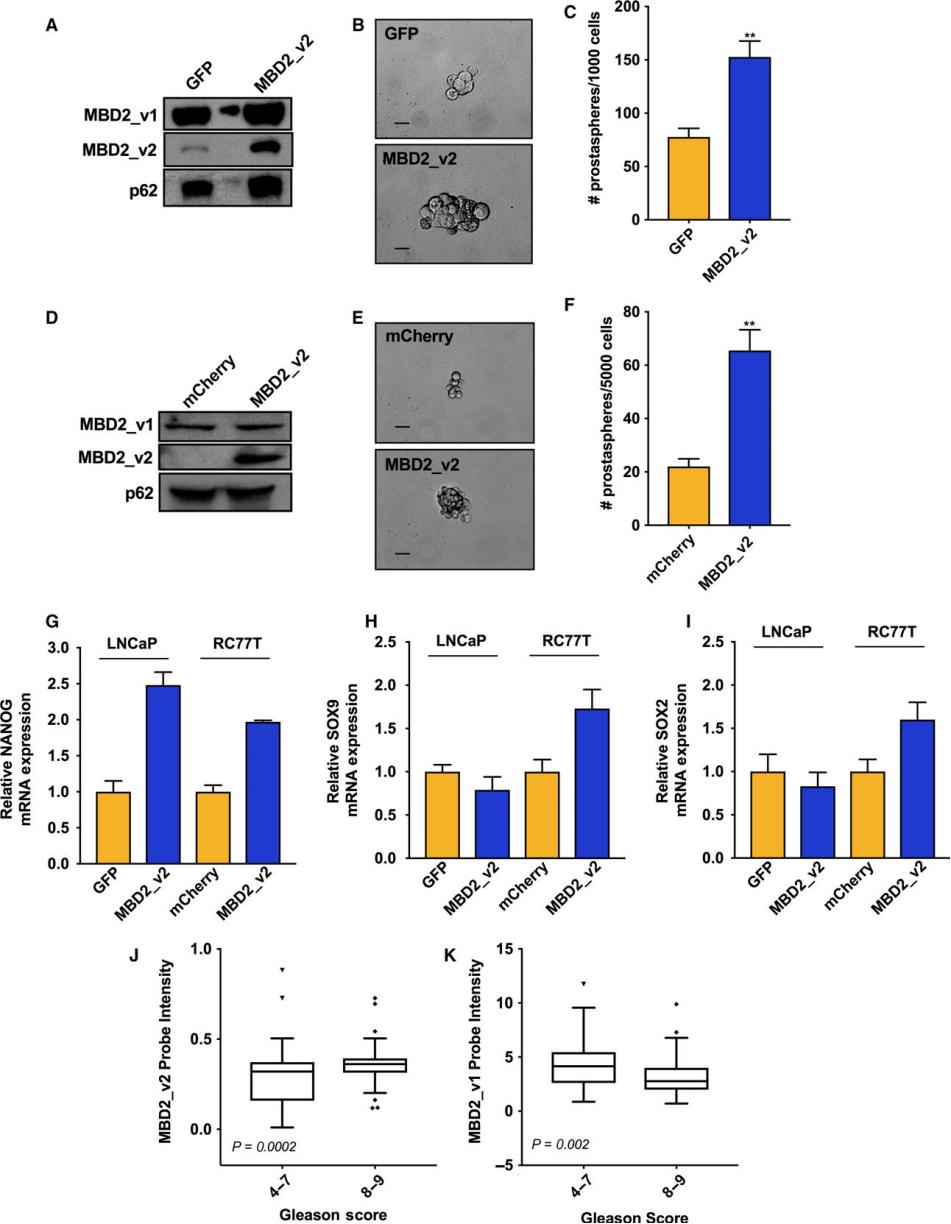
MBD2_v2 overexpression enhances prostasphere formation and is associated with high‐grade PCa. (A) Immunoblot measure of MBD2 isoforms in LNCaP cell line stably transduced with MBD2_v2 or GFP control expression vectors. (B,C) The effect of stable MBD2_v2 overexpression in LNCaP cells on prostasphere size and prostasphere numbers relative to GFP‐expressing LNCaP control cells. Bar = 1000 μm. (D) Immunoblot measure of MBD2 isoforms in RC77T cell line stably transduced with MBD2_v2 or mCherry control expression vectors. (E,F) The effect of stable MBD2_v2 overexpression in RC77T cells on prostasphere size and prostasphere numbers relative to mCherry‐expressing RC77T control cells. Three biological replicates were used in each prostasphere assay, which was performed twice (total of six biological replicates). Bar = 1000 μm. (G–I) Real‐time RT–PCR analysis was performed to measure the effect of MBD2_v2 stable overexpression on NANOG, SOX9, and SOX2 levels in LNCaP and RC77T cells. (J,K) PCa data sets compiled from Oncomine [GS 4‐7 (*n* = 171) and GS 8‐9 (*n* = 53)] were used to test if high MBD2_v2 or MBD2_v1 transcript expression associated with high GS. **P ≤ 0.01.

We proceeded to stably overexpress MBD2_v2 in LNCaP cells to assess the impact on CSCs via a prostasphere formation assay. Under nonattachment, serum‐free conditions overexpression of MBD2_v2 caused a significant increase in prostasphere numbers and an increase in prostasphere size relative to GFP‐expressing controls (Fig. 4A–C, Fig. S4). We subsequently performed the same experiment using the AA patient‐derived RC77T prostate cancer cell line, and the results were essentially the same (Fig. 4D–F, Fig. S4), underscoring that although a molecular phenotype may be enriched in PCa from AAM (i.e., TP53 wild‐type, IL‐6 derived from the environment), it is not exclusive to PCa from AAM. A report by Lu *et al*. (2014) details the mechanism whereby in human pluripotent stem cells (hPSCs), MBD2_v2 activates genes such as NANOG and SOX2. It is well known that SOX2 and NANOG directly interact and regulate self‐renewal of hPSCs and CSCs (Gagliardi *et al*., 2013; Jeter *et al*., 2016; Sarkar and Hochedlinger, 2013). We proceeded to test whether MBD2_v2 regulates the mRNA expression of SOX2 and NANOG in the context of PCa cells. SOX9 was also of interest to us based on a recent report that it fulfills a molecular function similar to SOX2, but may have a predominant role in therapy resistant PCa (Chen *et al*., 2016). The results complete a set of experiments providing evidence that exogenous IL‐6 treatment upregulates MBD2_v2 in TP53 wild‐type LNCaP and RC77T cells (Fig. 3) and that upregulated MBD2_v2 by stable overexpression in RC77T cancer cells upregulates NANOG, SOX2, and SOX9 (Fig. 4G–I). In LNCaP cells, only NANOG increased with MBD2_v2 overexpression (Fig. 4G). Perhaps giving some indication of differences for these two cell lines that had up to now in the course of our study appeared molecularly similar. Although based on the literature the cell function outcome will be the same: increasing any single one of these factors will likely promote the stemness phenotype (Chen *et al*., 2016; Gagliardi *et al*., 2013; Jeter *et al*., 2016; Sarkar and Hochedlinger, 2013).

Finally, analysis of Affymetrix microarray expression data sets, accessed via Oncomine (Rhodes *et al*., 2004), demonstrated that GS 8‐9 PCa express significantly higher levels of MBD2_v2 relative to GS 4‐7 PCa (Fig. 4J). Conversely, further analysis showed an inverse relationship between variant MBD2_v1 expression and PCa GS (Fig. 4K).

### 3.4 IL‐6 treatment decreased wild‐type TP53 protein in IL‐6 nonexpressing cells

**Figure 5:**
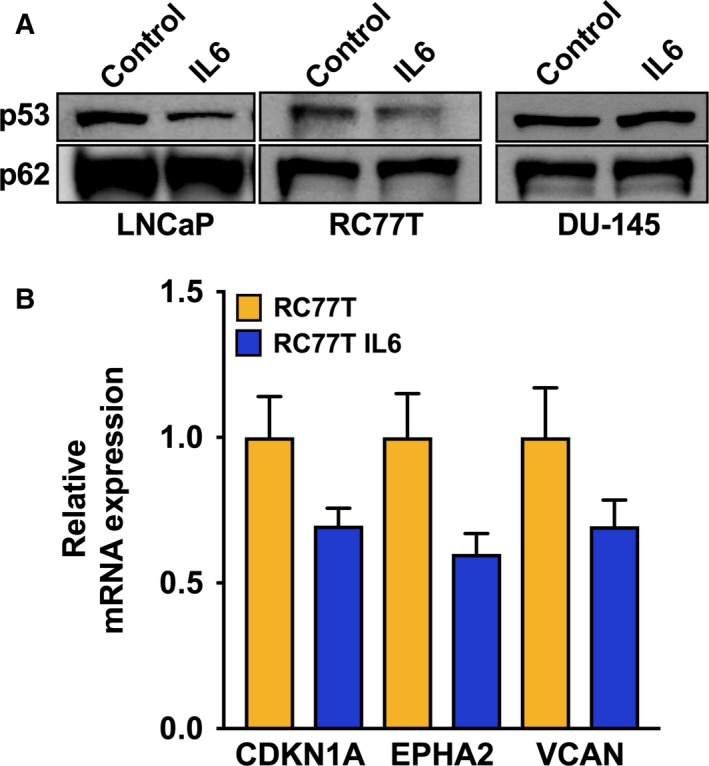
IL‐6 treatment downregulated wild‐type TP53 protein levels in non‐IL‐6 expressing PCa cell lines. (A) TP53 immunoblot analysis of IL‐6 nonexpressing, TP53 wild‐type RC77T, and LNCaP cell lines, and TP53 mutant DU‐145 cells, each treated with IL‐6 or vehicle control for 7 days. (B) Real‐time RT–PCR analysis of mRNA expression levels of known TP53‐regulated genes in RC77T cells treated with IL‐6 or vehicle control for 7 days. Cell culture treatment, protein harvest, and immunoblot analyses were carried out three times.

As described above, results of our RNA‐sequencing data analysis pipeline revealed that IL‐6 was at significantly higher levels in the noncancer, tumor‐adjacent tissue of AAM relative to PCa from AAM and tumor‐adjacent tissue from EAM. Also, although TP53 itself was not differentially expressed, the significant results from Upstream Regulator Analysis identified evidence for inactivation of wild‐type TP53 signaling in PCa from AAM (Fig. 1C,D). We predicted that these findings were related and hypothesized that microenvironment‐derived IL‐6, or exogenous IL‐6 treatment in culture, downregulates wild‐type TP53 protein levels in PCa cells. Wild‐type TP53 function is known to play a role in inhibiting the CSC phenotype (Chang *et al*., 2011; Ren *et al*., 2013), thus, this hypothesis is also relevant to IL‐6 promotion of CSCs. To test it, we measured the effect of IL‐6 treatment on TP53 levels using IL‐6 nonexpressing, TP53 wild‐type cell lines RC77T and LNCaP. Immunoblot analysis demonstrated that TP53 protein levels decreased in both RC77T and LNCaP cells treated with IL‐6 (Fig. 5A). Real‐time RT–PCR analysis validated that IL‐6 treatment did not induce TP53 mRNA level changes (data not shown). Also, for IL‐6 expressing DU145 cells, IL‐6 treatment had no effect on mutant TP53 levels (Fig. 5A). Lastly, by real‐time RT–PCR analysis we tested the effect of IL‐6 treatment on the expression of genes that are known to be regulated by wild‐type TP53 function using the RC77T cell line. We selected to test EPHA2 and VCAN because they are among the significant results from the RNA‐sequencing data analysis results associated with specimens from AAM in Fig. 1D, and because they are regulated by direct TP53‐DNA binding (Fischer, 2017). We also tested the more commonly studied TP53‐regulated factor cyclin‐dependent kinase inhibitor 1A (CDKN1A), otherwise known as p21. For all three genes, mRNA expression levels decreased with IL‐6 treatment (Fig. 5B).

## 4 Discussion

We began this investigation with RNA sequencing of PCa patient specimens, which produced new evidence of molecular diversity for high‐grade PCa associated with race. Our analysis identified race‐specific differential gene expression comparing tumor and noncancer adjacent tissue samples. Countering a previous report that PCa tumors lack IL‐6 expression (Yu *et al*., 2015), our RNA‐sequencing data analysis highlighted that PCa tumors from EAM, and by extension PCa cells, express relatively high levels of IL‐6. We measured IL‐6 expression across a diverse PCa cell line panel. DU145 and PC3 PCa cell lines expressed abundant IL‐6 mRNA, but IL‐6 was not detected in RNA harvested from LNCaP cells. Okamoto *et al*. (1997) reported similar findings based on measurement of IL‐6 protein secreted from these cell lines. Our panel also included RC77T and MDA‐PCa‐2b derived from AAM, and with this expanded panel, we observed that IL‐6 nonexpressing PCa cell lines – LNCaP, RC77T, and MDA‐PCa‐2b – are TP53 wild‐type. In contrast, IL‐6 expressing cell lines – DU145 and PC3 – are TP53 mutant. TP53 status in our diverse PCa cell line panel may reflect that TP53 mutations are less frequent in PCa from AAM relative to PCa from EAM (Huang *et al*., 2017; Lindquist *et al*., 2016).

The RNA‐sequencing data analysis results associated with AAM led us to test the potential for microenvironment‐derived, or exogenous IL‐6 to downregulate wild‐type TP53 protein in IL‐6 nonexpressing PCa cell lines. Immunoblot analysis showed that IL6 treatment caused a marked decrease in TP53 protein levels in TP53 wild‐type cell lines. In parallel, we observed that IL‐6 treatment had no effect on TP53 mRNA. Additional studies are needed to uncover the mechanism by which, wild‐type TP53 protein is downregulated by IL‐6 signaling in PCa cells. However, it is already reported that loss of wild‐type TP53 is required for cancer cell expression of the stem cell phenotype (Chang *et al*., 2011; Ren *et al*., 2013). Moreover, low TP53 wild‐type protein levels in PCa are associated with worse outcomes (Kluth *et al*., 2014), but it remains unclear whether higher IL‐6 levels in the adjacent stroma and serum of AAM correlate with low levels of wild‐type TP53 protein in PCa specimens from AAM.

We characterized the effect of IL‐6 on CSCs in our PCa cell line panel. Summarizing the results of these experiments, IL‐6 treatment of IL‐6 nonexpressing PCa cells elicited a significant, dose‐dependent increase in the number of CSCs. For IL‐6‐expressing PCa cell lines, adding IL‐6 to the media of IL‐6‐expressing cells did not increase the number of CSCs. These data suggest that in IL‐6‐expressing PCa cell line cultures the IL‐6 receptor population was saturated by endogenous IL‐6 levels. Our work underscores that previous, unsuccessful clinical trials appropriately assessed the significance of IL‐6 signaling in PCa progression, but may have failed in their approach to target IL‐6 or IL‐6 signal transduction factors JAK/STAT (Buchert *et al*., 2016; Dorff *et al*., 2010; Fizazi *et al*., 2012; Plimack *et al*., 2013). Zhong *et al*. (2016) propose that a higher affinity IL‐6 antibody with an extended half‐life will contribute to solving the issue. On the other hand, more thorough understanding of downstream IL‐6 signaling mechanisms driving PCa CSCs could provide insights for improved PCa treatment strategies. Our finding that IL‐6 signaling upregulates expression of MBD2_v2, to support and promote expansion of the CSC niche in PCa, opens a novel avenue for research. CSCs are identified in patient tumors and tumor‐derived cell line cultures as a subfraction of self‐renewing, tumor‐initiating PCa cells that also give rise to drug resistance and metastatic recurrence (Maitland and Collins, 2008). The insight for us to test the effect of exogenous IL‐6 treatment on MBD2_v2 expression, and subsequently observe that upregulated MBD2_v2 increases PCa CSCs, is based on results of our investigation into how ROS signaling promotes malignant transformation and the stem cell phenotype in triple negative breast cancer cells (Bao *et al*., 2017). Our current study identified that MBD2_v2 sustains PCa CSCs. Furthermore, a pro‐inflammatory signaling environment (i.e., exogenous IL‐6) induces MBD2_v2 expression that drives expansion of the CSC population in TP53 wild‐type PCa cells. With these two studies, we have uncovered a mechanism implicated in two cancer types that disproportionately impact African Americans.

We intend to pursue studies to uncover further mechanistic insights surrounding how MBD2_v2 expression is regulated by IL‐6 in PCa. However, we can hypothesize that the mechanism by which MBD2_v2 functions to maintain and promote the generation of CSCs is similar to the mechanism described for hPSCs. MBD2_v2 is one of two alternative mRNA splicing variants for the epigenetic reader MBD2 gene and in hPSCs MBD2_v1 binds methylated CpG promoter sequence and recruits the Nucleosome Remodeling and Deacetylase (NuRD) corepressor complex to silence transcription of pluripotency genes and promote cellular differentiation (Lu *et al*., 2014). MBD2_v2 binds the same promoter sequences, but lacks the domain required to recruit the NuRD complex; and upregulated MBD2_v2 displaces MBD2_v1 to promote stem cell phenotypes (Lu *et al*., 2014). Analysis using the Oncomine gene expression microarray database (Rhodes *et al*., 2004) showed that high MBD2_v2 expression in patient tumors correlated with high‐grade PCa and that high MBD2_v1 expression correlated with low‐grade PCa. We do not yet have preliminary insight as to whether MBD2_v2 is differentially expressed in PCa from AAM relative to EAM. Public gene expression data sets are lacking in AAM specimens, and despite having achieved approximately 100 million high‐quality paired end reads per sample, MBD2_v2 mapped read counts were below the detection threshold in our RNA‐sequencing data. This underscores the challenge of using genomewide RNA sequencing to analyze specific mRNA splicing variants (Mehta *et al*., 2016).

Based on data from previous studies with a focus on PCa tissue, there appears to be no association between IL‐6 levels in cancer cells and high‐grade PCa (Pencik *et al*., 2015; Powell *et al*., 2013). Herein, we report that AAM with high‐grade cancer have significantly higher IL‐6 expression in the tumor microenvironment. Furthermore, AAM have higher circulating levels of IL‐6 relative to EAM (Maurice *et al*., 2017), and AAM are more likely to advance to higher grade disease (Maurice *et al*., 2017). Thus, further research to define the signaling mechanism for induction of MBD2_v2 expression in PCa, by IL‐6 derived from the microenvironment, may be particularly relevant for AAM.

## 5 Conclusion

In conclusion, the results of the current study contribute to characterizing gene expression patterns in high‐grade PCa and noncancer tissues from EAM and AAM. The results advance molecular understanding of how IL‐6 signaling promotes the CSC phenotype in PCa cells derived from EAM and AAM. Continued research is warranted to realize how these new insights for CSC biology can be exploited to overcome PCa race disparities and improve outcomes for all men.

## Author contributions

AB and IP involved in conception and design. GD, CL, AB developed methodology. EAT, BB, WS, AB acquired data. EAT, BB, CM, GD, CL, JDC, IP, AB were involved in analysis and interpretation of data (e.g., statistical analysis, biostatistics, computational analysis). EAT, BB, GD, CL, WS, JDC, IP, AB participated in writing, review, and/or revision of the manuscript. Administrative, technical, or material support provided by CM. Study was supervised by AB.

## Supporting information


**Fig. S1.** Assessment of isolated RNA and library preparation quantity and quality by spectrophotometry and TapeStation analysis.
**Fig. S2.** Comparison of mean FPKM read counts from RNA‐sequencing analysis and mean expression values from DASL microarray analysis of patient specimens.
**Fig. S3.** Top ranked significant differentially expressed genes (p < 0.05), based on analysis of RNA‐sequencing data from PCa and noncancer adjacent tissues as a function of race.
**Fig. S4.** Additional representative images comparing prostaspheres from control and MBD2_v2 overexpressing cell lines.
**Table S1.** Gleason Score for each PCa sample used in RNA‐sequencing analysis.
**Table S2.** Significant results from pathway enrichment analysis of differentially expressed genes.
**Table S3.** Data from FACS‐based analysis of the cancer stem‐like cell fraction in IL‐6‐treated and nontreated PCa cell lines.

**Data S1.** Materials and methods
