# Supplementary material for: Exogenous IL‐6 induces mRNA splice variant MBD2_v2 to promote stemness in TP53 wild‐type, African American PCa cells
Source: Mol Oncol. 2018 May 24;12(7):1138–52. doi: 10.1002/1878-0261.12316 (PMC6026877; doi:10.1002/1878-0261.12316)
Supplement: Supplementary file 1 — Fig. S1. Assessment of isolated RNA and library preparation quantity and quality by spectrophotometry and TapeStation analysis. Fig. S2. Comparison of mean FPKM read counts from RNA‐sequencing analysis and mean expression values from DASL microarray analysis of patient specimens. Fig. S3. Top ranked significant differentially expressed genes (p < 0.05), based on analysis of RNA‐sequencing data from PCa and noncancer adjacent tissues as a function of race. Fig. S4. Additional representative images comparing prostaspheres from control and MBD2_v2 overexpressing cell lines. Table S1. Gleason Score for each PCa sample used in RNA‐sequencing analysis. Table S2. Significant results from pathway enrichment analysis of differentially expressed genes. Table S3. Data from FACS‐based analysis of the cancer stem‐like cell fraction in IL‐6‐treated and nontreated PCa cell lines. [file MOL2-12-1138-s001.pdf]

trinean

DropSense REPORT

RNA Quality and Quantity Estimation by Spectrophotometry				
Sample ID (deidentified)	A260 Concentration (ng/ul)	A260/A280	Approximate RNA Yield (ug)	Plate Location
1014-C17	144.8	2.10	7.96	A1
1014-C9	51.88	2.00	2.85	B1
1193-C15	73.19	2.03	4.03	C1
1193-C12	109.49	2.06	6.02	D1
3139-18	50.62	2.01	2.78	E1
3139-12	111.65	2.03	6.14	F1
1136-11	211.21	2.01	11.62	G1
1136-20	827.03	1.99	45.49	H1
2249-25	44.89	2.08	2.47	A2
2249-14	177.75	2.06	9.78	B2
487-31	157.01	2.06	8.64	C2
487-33	393.67	2.03	21.65	D2
881-B26	132.91	2.00	7.31	E2
881-B19	215.58	2.03	11.86	F2
8047-C36	76.67	2.01	4.22	G2
8047-C11	153.01	2.04	8.42	H2
334-A7	384.74	1.98	21.16	A3
334-A24	127.88	1.99	7.03	B3
413-15	81.89	2.00	4.50	C3
413-24	115.79	2.02	6.37	D3
3365-B5	226.03	2.05	12.43	G3
3365-B17	78.59	2.04	4.32	H3
647-A7	229.95	2.05	12.65	A4
647-A20	104.27	2.03	5.73	B4
3869-B12	75.61	1.98	4.16	C4
3869-B22	137.24	2.02	7.55	D4
376-8	233.33	2.06	12.83	E4
376-9	443.88	2.07	24.41	F4
6053-C13	404.66	2.03	22.26	G4
6053-C14	355	2.04	19.53	H4
3309-22	230.22	2.03	12.66	A5
3309-40	137.48	2.02	7.56	B5
blank	0	-		

High Sensitivity D1K ScreenTape®

Controller Notes

Bollig-Fischer. Nugen FFPE RNA libraries. 1ul product run.

Gel Image

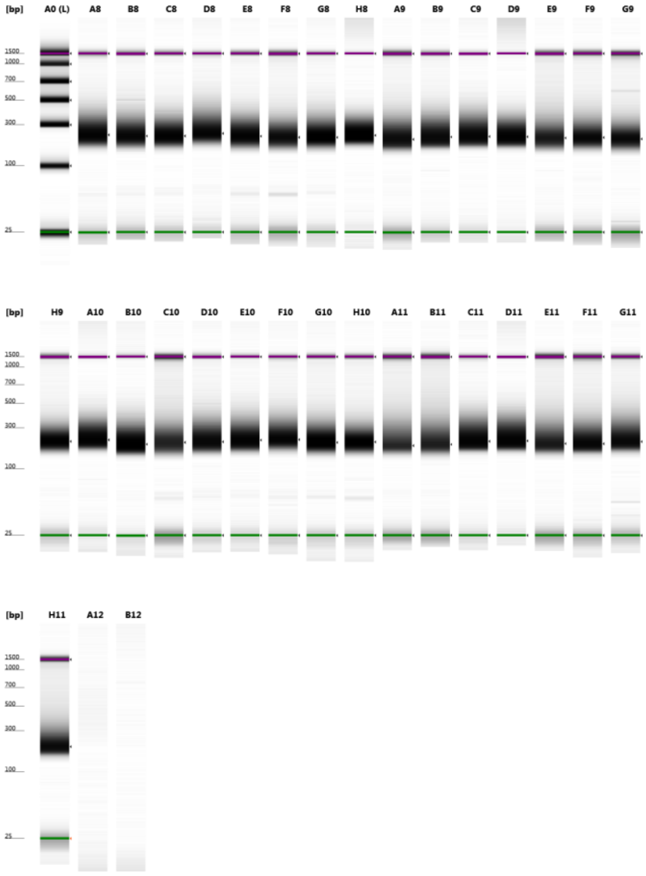

2200 TapeStation Software (A.01.03)

Supplementary Figure S1. ***(Left panel)*** Quantity and quality of RNA isolated from FFPE samples was estimated by spectrophotometry analysis using the Trinean DropSense (PerkinElmer, Waltham, MA). ***(Right panel)*** The state of each sample library preparation was assed prior to sequencing using the TapeStation (Agilent, Santa Clara, CA). Here the quality in library preparation is demonstrated by the consistency in fragment size and appropriate yield. Lane order, starting top left lane 2, correspond to listing in left panel.

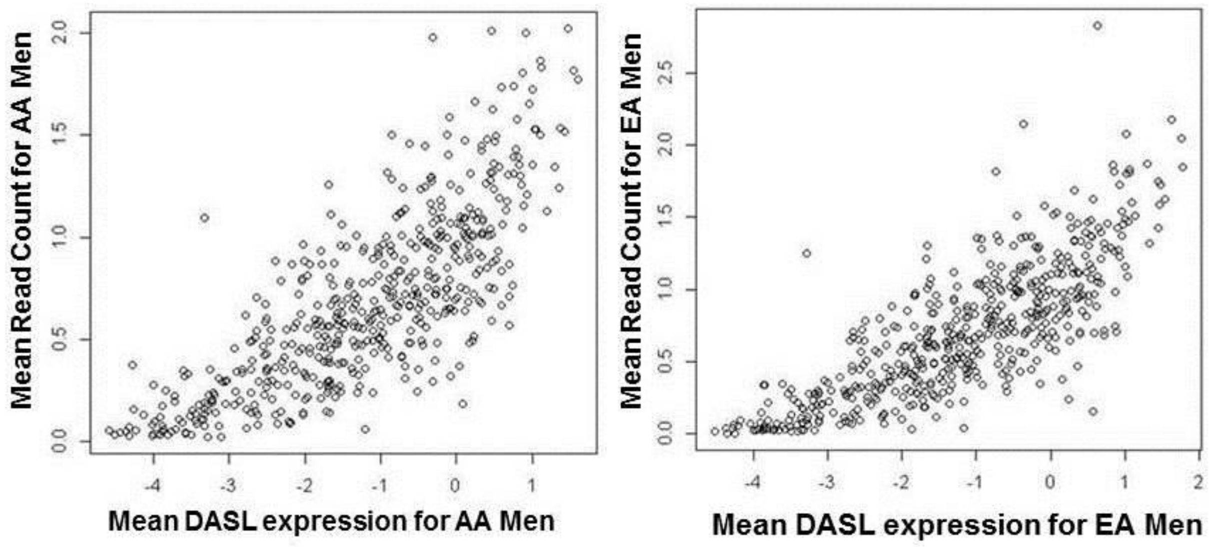

Supplementary Figure S2. Comparison of mean values for log (base 10) FPKM read counts from RNA-sequencing analysis and mean expression values from DASL microarray analysis. Sequencing data represents 16 PCa specimens from 8 African American (AA) men and 8 European American (EA) men, all with Gleason score (GS)≥7(4+3). DASL gene expression data is accessible via Gene Expression Omnibus GSE41969 and represents 95 AA and 134 EA PCa specimens. The sample sets were non-overlapping and analysis was limited to genes measured by the microarray (n=512). A test of non-parametric (Spearman) correlation between measurements from the two technologies yields a correlation of 0.805 for AA men and 0.811 for EA men.

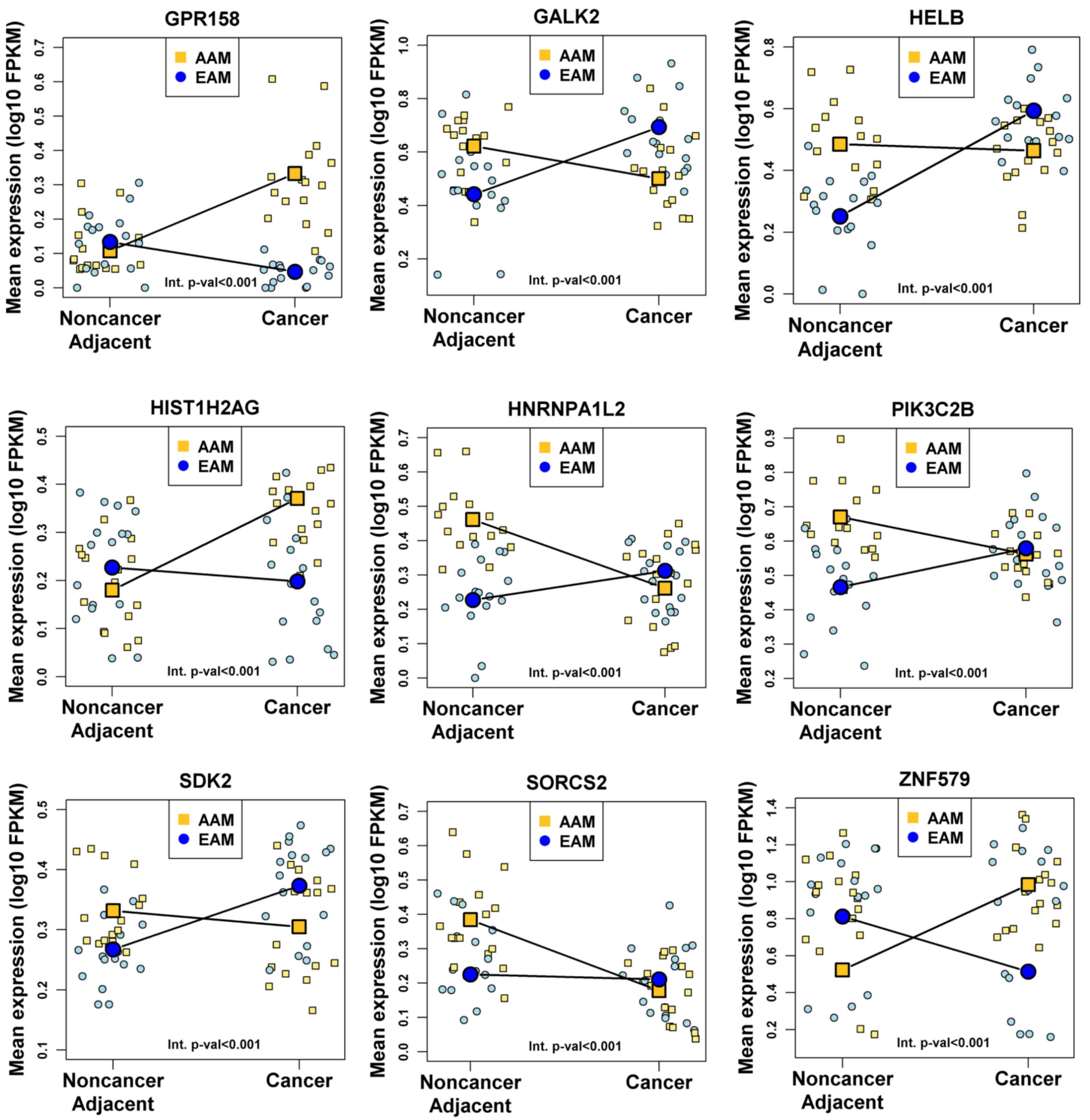

Supplementary Figure S3. Top 9 most significant differentially expressed genes (p<0.05), based on analysis of RNA-sequencing data from PCa and noncancer adjacent tissues as a function of race.

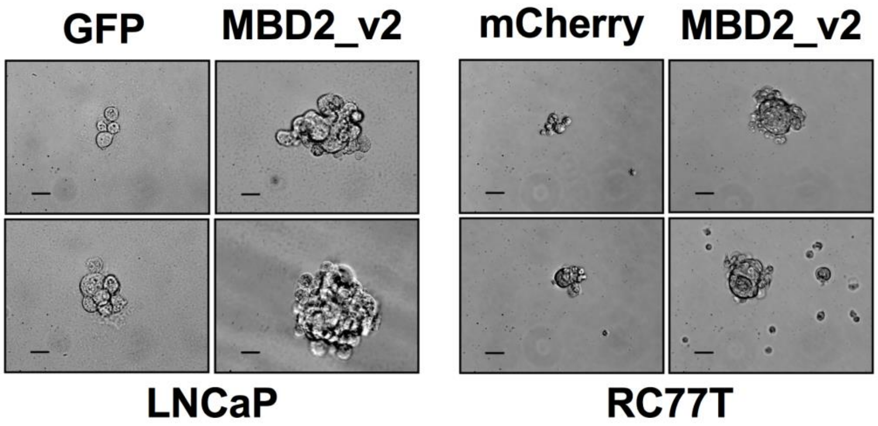

Supplementary Figure S4. Additional representative images demonstrating the effect of stable MBD2_v2 overexpression in LNCaP and RC77T cells on prostasphere size relative to GFP or mCherry expressing control cells.
Bar = 1,000 µm.

Patient No.	Race	Grade
1	AA	8
2	AA	8
3	AA	9
4	AA	9
5	AA	8
6	AA	9
7	AA	8
8	AA	8
9	C	7 (4+3)
10	C	7 (4+3)
11	C	7 (4+3)
12	C	7 (4+3)
13	C	8
14	C	8
15	C	7 (4+3)
16	C	7 (4+3)

Supplementary Table S1. Gleason Score/Grade for each PCa sample used in RNA-sequencing analysis. RNA sequencing was performed on matched high grade [GS≥7(4+3)] prostate tumor and adjacent nonmalignant specimens from 16 patients (8 AAM and 8 EAM.

Supplementary Table S2. The Enrichr tool was used to identify significantly over-represented KEGG pathways in the results of RNA-sequencing data differential analysis comparing tumor versus normal gene expression as a function of race. Genes from our analyzed dataset that are within each significant pathway are listed, including cytokines IL6 and TGFB1.

KEGG Pathway	Overlap	P-value	Genes from analyzed dataset in pathway
Cytokine-cytokine receptor interaction_Homo sapiens_hsa04060	29/265	0.003	CNTF;CD40;IFNA7;IFNA1;IL26;FLT3;IL23R;PDGFB;IL18RAP;CCR8;TNFRSF17;IL12A;PDGFRB; PDGFRA;XCR1;TGFB1;CCL21;IL11RA;TNFRSF18;FLT3LG;INHBB;TNFRSF1B;IL6;IL23A;XCL1; TNFRSF25;IL7R;TNFRSF21;IL9R
Neuroactive ligand-receptor interaction_Homo sapiens_hsa04080	27/277	0.020	PTGFR;RXFP4;PLG;HTR2A;GRPR;RXFP2;GRM1;GRM3;GHRHR;HRH1;CCKAR;NPBWR2;DRD2;GRIA3;NTSR2;GABRA2;UTS2R;CHRNB4;TAAR8;GCGR;GABRA3;TACR1;GRIN2C;HCRTR2; P2RX2;MC5R;F2RL3
Inflammatory bowel disease (IBD)_Homo sapiens_hsa05321	9/65	0.022	SMAD2;IL6;TGFB1;IL18RAP;IL23A;IL23R;IL12A;FOXP3;HLA-DPA1
Calcium signaling pathway_Homo sapiens_hsa04020	19/180	0.022	PDGFRB;PRKCG;PDGFRA;PTGFR;ATP2B3;TACR1;HTR2A;ATP2B1;GRIN2C;CACNA1F;GRPR; GRM1;SLC8A2;HRH1;PPP3CC;CCKAR;P2RX2;PLCG2;CACNA1S
Pathways in cancer_Homo sapiens_hsa05200	35/397	0.035	FLT3;PDGFB;LAMC2;HIF1A;ETS1;ADCY5;FGF4;FGF5;FGF6;WNT11;CASP8;PLCG2;VHL;WNT1;PRKCG;PDGFRB;SMAD2;STAT5B;PDGFRA;EGLN2;TGFB1;LAMB2;FLT3LG;GNG12;IL6;CDK6; RAD51;CCNE1;COL4A3;COL4A6;RARB;COL4A5;ITGA6;FGF12;F2RL3

Supplementary Table S3. FACS data collected for IL6-treated and non-treated PCa cell lines with sorting of the cancer stem-like cell fraction based on positive status of three surface markers, CD44, CD133, and EPCAM. Based on analysis of 3 independent replicates, the values under the Mean Percentage (%) column represent the triple marker-positive cell population fraction of the mean total live cell count. For each cell line, fold-change represents the percentage of triple marker positive cells in IL6 treated cultures relative to the percentage in the vehicle control condition. SEM, standard error of mean.

PCa Cell Line	Sample Treatment	Mean Total Cell Count	Mean % single/live cells CD44+/CD133+/EPCAM+	Fold-Change	SEM	p-value
**LNCaP**	IL6	519923	3.718	3.3	0.5115	0.02
	Control	1054734	1.132	1.0	0.2710	
**RC77T**	IL6	505971	0.012	2.5	0.0015	0.02
	Control	671333	0.005	1.0	0.0007	
**PC3**	IL6	490014	0.059	1.6	0.0238	0.93
	Control	449287	0.037	1.0	0.0144	
**DU-145**	IL6	600000	0.029	1.1	0.0087	0.97
	Control	612667	0.026	1.0	0.0072	
