# Supplementary material for: Exogenous IL‐6 induces mRNA splice variant MBD2_v2 to promote stemness in TP53 wild‐type, African American PCa cells
Source: Mol Oncol. 2018 May 24;12(7):1138–52. doi: 10.1002/1878-0261.12316 (PMC6026877; doi:10.1002/1878-0261.12316)
Supplement: Supplementary file 2 — Data S1. Materials and methods [file MOL2-12-1138-s002.pdf]

Supplementary Materials and Methods

Cell lines, culture conditions and treatments

The established PCa cell line MDA-PCa-2b was newly purchased from ATCC (ATCC, Manassas, VA) for this study. The established cell lines LNCaP, PC3 and DU-145 were acquired from the Biobanking and Correlative Sciences Core (BCSC) at the Karmanos Cancer Institute where they were passaged and authenticated by short tandem repeat (STR) analysis using the PowerPlex(r) 16 system (Promega, Madison, WI) immediately prior to use in this study. RC77T PCa cell line was established and provided to us by Dr. Clayton Yates (1). LNCaP, PC3 and DU-145 cells were maintained in 10% FBS RPMI-1640 media containing 50 µg /mL gentamycin at 37 °C, 5% CO_2. RC77T cells were seeded on plates coated with FNC Coating Mix™ (ATHENA, Baltimore, MD, catalogue number 0407) in Gibco keratinocyte-SFM media (Thermo Fisher, Waltham, MA, catalogue number 10724-011), supplemented with EGF and BPE, with 2% FBS immediately added to each plate after splitting. FBS containing media was replaced by keratinocyte-SFM media, supplemented with EGF and BPE, 24 hrs after splitting or seeding and RC77T cells were maintained at 37 °C, 5% CO_2. MDA-PCa-2B cells were maintained in 10% FBS F-12K media (Corning Inc., Corning, NY, catalogue number 10-025-cv,) containing 1% penicillin/streptomycin supplemented with 25 ng/mL cholera toxin, 10 ng/mL EGF, 0.005 mM phopsho-ethanolamine, 100 pg/mL hydrocortisone, 45 nm selenious acid at 37 °C, 5% CO_2. IL-6 was purchased from BD Biosciences (Franklin Lakes, NJ). STAT3 inhibitor cryptotanshinone (CTS) was purchased from Sigma-Aldrich (St. Louis, MO, catalogue number C5624-5MG). The IL6 receptor inhibitor drug, Tocilizumab (Genentech, South San Francisco, CA), was from the Karmanos Cancer Institute pharmacy.

Immunoblot analysis

Nuclear extracts were obtained using the NE-PER Nuclear extraction kit (Thermo Fisher). Immunoblot analysis was used to detect the relative levels of MBD2, total STAT3, and pSTAT3, proteins Briefly, 50 ug of total protein from each sample were loaded and separated on 10% SDS PAGE gels, and then transferred to a nitrocellulose membrane using Mini Trans-Blot Electrophoretic Transfer Cell (Bio-Rad, Hercules, CA). Membranes were probed with primary antibodies following supplier recommendation and secondary peroxidase-conjugated antibodies (anti-mouse or rabbit). Primary antibodies included: α-MBD2 (Bethyl Laboratories, Montgomery, TX, catalogue number A301-633A); TP53 (Thermo Fisher Scientific, Waltham, MA, catalogue number MS105P0); α-STAT3 and α-pSTAT3 (Abcam, Cambridge, UK, catalogue numbers ab119352 and ab76315); and mouse monoclonal α-nucleoporin P62 (Sigma-Aldrich, St. Louis, MO, catalogue number 610497) for nuclear extract controls.

Semi-Quantitative RT-PCR using TaqMan assays

RNA was extracted from cells using the Qiagen (Valencia, CA) RNeasy kit. RNA was converted into cDNA via a reverse transcription reaction using High-Capacity RNA-to-cDNA Kit from Thermo Fisher Scientific (Waltham, MA). TaqMan gene expression master mix and TaqMan assays for IL6 and MBD2_v2 (catalogue numbers Hs00985639 and Hs00210557) were from Thermo Fischer Scientific (Waltham, MA). SYBR Green master mix was from applied bioysystems, and primers were purchased from Integrated DNA Technologies using Harvard Primer Bank primer sets for CDKN1A, VCAN, EPHA2, SOX2, NANOG, SOX9, and (PrimerBank IDs 310832423c2, 255918075c1, 296010835c1, 325651854c2, 153945815c3 and 182765453c1). For these experiments, 20 uL reactions were run in 96-well plates using 100-1000ng cDNA. Reactions were run in triplicate using the StepOnePlus Real-Time PCR System (Applied Biosystems, Foster City, CA). β-Actin (cat#Hs99999903) was used as the reference gene in all experiments. Relative quantification was calculated using the ΔΔCt method (2).

Viability Assays

For viability assay, cells were seeded at 3000 cells/well in 96-well plates, and incubated at 37 °C, 5% CO_2 for 24 hrs. Cells were then treated with 0.5 ng/mL, 10 ng/mL or no IL6 from BD Biosciences (Franklin Lakes, NJ), and incubated for 7 days at 37 °C, 5% CO_2. Following IL6 treatment, MTT assays or ATP assays were performed using the CellTiter-Glo® Luminescent Cell Viability (Promega , Madison, WI, catalogue number G7571) or Vybrant® MTT Cell Proliferation (Life technologies, Carlsbad, CA, catalogue number V13154)

Assay Kits, respectively. Cell viability is shown as percentage (%), comparing mean cell viability for IL6 treated to non-treated negative control samples.

References

1. Theodore S, Sharp S, Zhou J, Turner T, Li H, Miki J, et al. Establishment and characterization of a pair of non-malignant and malignant tumor derived cell lines from an African American prostate cancer patient. International journal of oncology. 2010;37:1477-82.
2. Bookout AL, Cummins CL, Mangelsdorf DJ, Pesola JM, Kramer MF. High-throughput real-time quantitative reverse transcription PCR. Curr Protoc Mol Biol. 2006;Chapter 15:Unit 15 8.
